# STATE-LEVEL INITIATIVES FOR DIRECT CARE WORKERS

**DOI:** 10.1093/geroni/igad104.0977

**Published:** 2023-12-21

**Authors:** Bailee Brekke

**Affiliations:** Miami University, Oxford, Ohio, United States

## Abstract

As problems surrounding recruitment and retention of direct care workers continue to worsen, changes have been made at both the state and national level to accommodate for these challenges. This paper provides a literature review of various state-level initiatives and innovations aimed at improving recruitment and retention strategies for direct care workers in both facility-based long-term care and home and community-based long-term services across the country. Research revealed three themes, 1) strategies to improve wages and benefits, 2) education and training, and 3) community support. To improve wages and benefits, many states increased compensation and benefits for DCWs with the implementation of wage pass-throughs, minimum wage increases, and earmarked funds. Education and training efforts were identified as another way to enhance recruitment and retention efforts for the direct care workforce, such as through credential and certification opportunities for workers, as well as standardizing current training requirements. Lastly, increasing community support was identified through advocacy and research efforts specifically aimed at understanding and supporting DCWs. This study seeks to inform policymakers and providers of current initiatives that can be used to address the ongoing workforce crisis.

